# A comparison of fibrin glue versus suture for anchoring fat pedicles in transconjunctival lower eyelid blepharoplasty

**DOI:** 10.1016/j.jpra.2025.01.018

**Published:** 2025-01-31

**Authors:** Ryo Kikuchi, Kanjanawan Meeprasertsagool, Duangmontree Rojdamrongratana, Tomoyuki Kashima

**Affiliations:** a Oculofacial Clinic Group, Tokyo, Japan; b Kameda Medical Center, Chiba, Japan; c Bangrajun Hospital, Singburi, Thailand; d Thammasat University, Bangkok, Thailand

**Keywords:** Lower eyelid blepharoplasty, Fibrin glue, Fat repositioning, Skin sutures

## Abstract

**Introduction:**

Lower eyelid blepharoplasty involves several surgical techniques. In conventional procedures, fat pedicles are temporarily sutured with the external skin or internally fixed with the periosteum to anchor the fat. In this study, we compare a new method, which requires no sutures, of anchoring fat using fibrin glue to the conventional way of temporarily suturing with external skin.

**Methods:**

We retrospectively collected data to identify 127 patients who underwent transconjunctival lower eyelid blepharoplasty with fat transposition to correct tear troughs. The patients were divided into two groups: 55 patients had the repositioned fat anchored with sutures and 72 patients had the fat anchored using fibrin glue. We focused on the score of postoperative complications multiple times.

**Results:**

In terms of skin discoloration, 57.14 % of the patients in the suture group exhibited Grade 2 changes and 42.85 % reached Grade 3. In contrast, only 36.36 % of the patients in the fibrin glue group reached Grade 2, with the majority (54.54 %) showing mild Grade 1 discoloration, and no cases reaching Grade 3. For eyelid tension, no patients in the fibrin glue group experienced severe Grade 3 tension, compared to 2.04 % of the patients in the suture group. Additionally, severe Grade 3 contusions appeared in 71.42 % of the patients the suture group but only in 36.36 % of the patients in the fibrin glue group.

**Conclusions:**

Among the patients who underwent transconjunctival lower eyelid blepharoplasty with fat transposition, the use of fibrin glue produced substantially lowered the severity of skin discoloration and reduced hematoma tension compared to sutures.

## Introduction

Lower eyelid blepharoplasty involves several surgical techniques without an established universal approach. The goal of lower eyelid blepharoplasty is to restore a youthful appearance to the lower eyelid while preserving its function. These procedures can be performed using either a transconjunctival or transcutaneous incision. Transconjunctival orbital fat repositioning was first reported by Goldberg.^1^^,^^2^ Transconjunctival lower eyelid blepharoplasty can minimize the risks of complications, such as lower eyelid retraction or postoperative bruising and scarring.^3^ The most commonly used method for transconjunctival lower eyelid blepharoplasty is pedicled fat transposition, where the orbital fat is preserved and transposed over the inferior orbital rim. This technique can correct tear trough deformity.^4^ In the conventional procedures, fat pedicles are temporarily sutured with external skin or internally fixed with periosteum to anchor the fat.^5^ However, the patient's quality of life is diminished until the skin sutures are removed postoperatively, as the sutures remain in the cheek. Visible sutures after lower eyelid blepharoplasty can impact the patients’ daily comfort and confidence by prolonging their social downtime. In this research, we compared a new method of anchoring fat using fibrin glue, without sutures, to the conventional method of temporarily suturing with external skin.

## Methods

We retrospectively collected data from two oculofacial clinics in Japan, namely Oculo Facial Clinic Tokyo and Shinmaebashi Kashima Oculoplastic Clinic, to identify 127 patients who underwent transconjunctival lower eyelid blepharoplasty with fat transposition to correct tear troughs between April 2017 and April 2022. All patients had no history of blood disease, antithrombotic medications, filler injections, or lower eyelid surgery. The patients were divided into two groups: 55 patients had the repositioned fat anchored with sutures and 72 patients had the fat anchored using fibrin glue.

The primary endpoint, defined as the time to social downtime recovery, was assessed based on the duration until patients could resume social activities without visible signs of surgery, specifically without external sutures.

All participants were informed of the procedures to be used and possible complications, after which they signed the written informed consent. This study was approved by the Ethics Committee of Oculo Facial Clinic Tokyo, Japan, on July 28, 2023. This study adhered to the ethical principles outlined in the Declaration of Helsinki, as amended in 2013.

### Surgical technique

All patients underwent the surgical procedure under monitored anesthesia care with local anesthesia in the supine position. The midcheek was infiltrated with 2 % lidocaine and 1:100,000 epinephrine, and 2 ml of local anesthesia was administered at each site. An eyelid retractor was used to retract the lower eyelid, and Westcott scissors were used to create a transconjunctival incision along the horizontal length of the lower eyelid, approximately 2 mm below the inferior edge of the inferior tarsus. Silk 5–0 was used to retract the conjunctiva and lower eyelid retractors. The orbital septum was dissected and opened to expose the orbital fat. Caution was exercised while exploring the medial and central fat pads to avoid injuring the inferior oblique muscle. The lateral fat pad was removed. The raspatories were used to release the orbicularis retaining ligament from the periosteum of the orbital rim, and then we created a pocket in the periosteum. As the ligament tends to recur in the medial side, we attempted to detach the nasal bone when we created the pocket. Care was taken not to injure the infraorbital nerve at the time. Monocryl 3–0 was used to suture the medial and central fat pads, then the two fad pads were transposed over the orbital rim, placed into the predissected pocket, and distributed evenly within it.

After completing all the steps, the patients were divided into two groups. The first group underwent fat transposition to the premaxillary area, followed by tying the suture externally at the skin. In the second group, fat transposition to the premaxillary area was also performed, and the suture was passed through the skin. The fibrin glue was then applied to the area where the fat was transposed. Subsequently, the suture was cut. In both groups, the transconjunctival incision site was closed without suturing.

### Study procedures

We focused on three dimensions of postoperative complications (Table 1), namely changes in skin color, eyelid contusion (measured by the range of contusion area), and skin tension evaluated by comparing marginal reflex distance 2 (MRD-2) before and after the operation. The severity of the complications was assessed on a scale of 0 to 3 multiple times following surgery, including at 1 week, 2 wk, 1 month, 2 months, and 3 months after the operation.

**Table 1:** Scoring chart.

Score	0	1	2	3
Color	No change	Yellow	Purple	Red or black
Range	No change	Only lower eyelid	Beyond the orbital margin or subconjunctival hemorrhage	Extend to the upper eyelid
Eyelid tension	No tension	Mild tension (MRD-2 change is 1–2 mm compared to preoperation)	moderate tension (MRD-2 change is ≧3 mm compared to preoperation)	Severe tension (MRD-2 change is ≧3 mm compared to preoperation and it is difficult to open eyelid)

**Figure 1:**
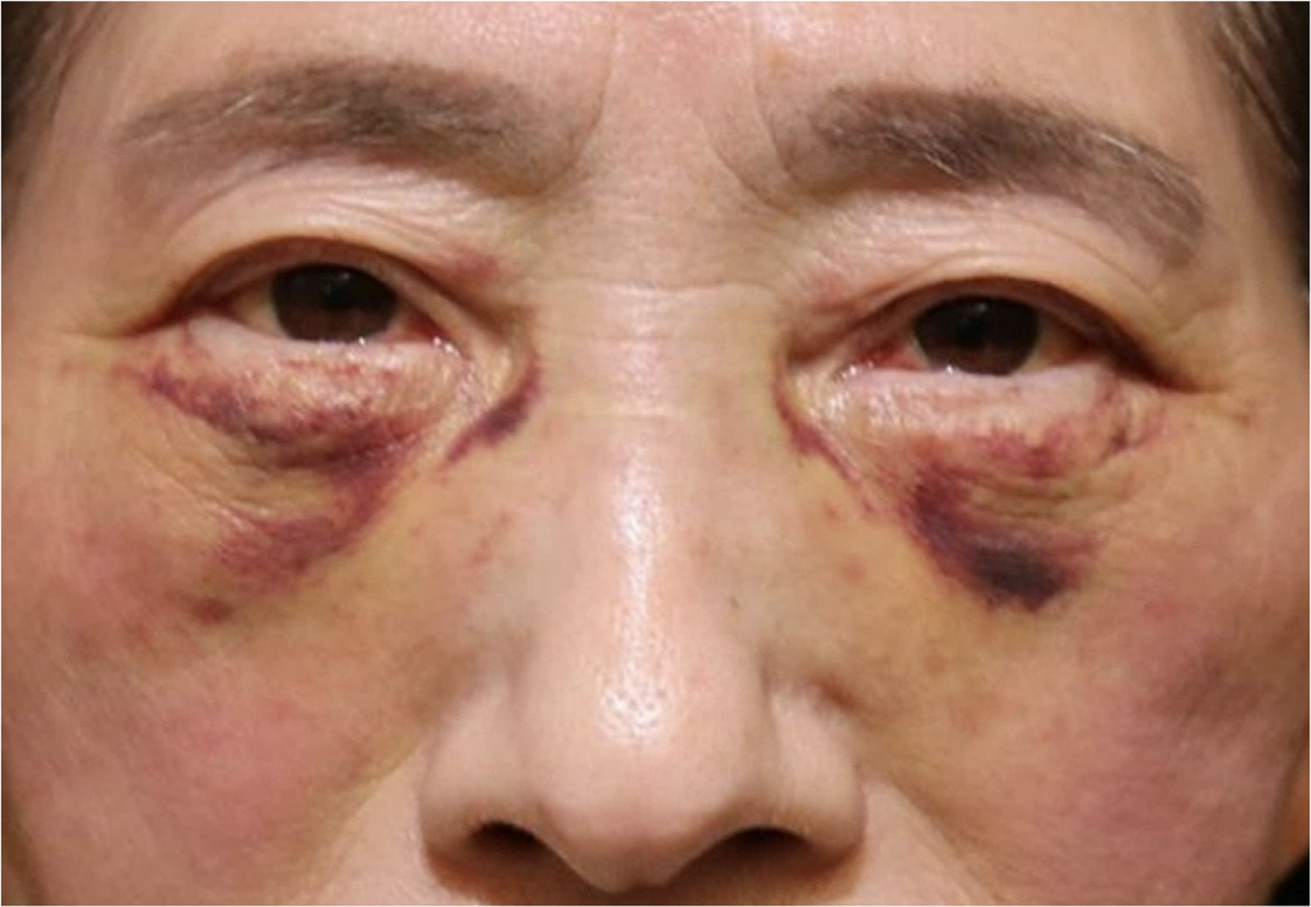
Example of grading using the patient's facial photograph.

In detail, the first symptom that we evaluated was the change in skin color after the operation, which was graded on three levels: Grade 1 for yellow, Grade 2 for purple, and Grade 3 for red/black. Another complication was eyelid contusion, which was graded on a three-point scale of 1 to 3: Grade 1 for a skin defect only at the lower eyelid, Grade 2 for beyond the orbital margin or having a subconjunctival hemorrhage, and Grade 3 for extending to the upper eyelid. The last symptom was the change in skin tension at the surgical site, which was recorded by comparing MRD-2 before and after surgery and graded into three severities: Grade 1 mild tension (MRD-2 change is 1–2 mm), two moderate tension (MRD-2 change is ≧3 mm), and three severe tension (MRD-2 change is ≧3 mm and making it difficult to open the eyelid). MRD-2 was measured as the distance from the center of the pupil to the lower eyelid margin in the primary gaze. MRD-2 distances measured in Image J ver. 1.53. These evaluations were assessed by a single evaluator who was blinded to the surgical procedure. For example, in Figure 1, the color of the birthmark was black (Grade 3), affected area extended to the upper eyelid (Grade 3), and change in MRD-2 was mild (Grade 1). Additionally, we evaluated postoperative complications.

### Statistical analysis

In the primary analysis, the outcomes of the surgery were described as the proportion of severity of each complication dimension at each follow-up time, which were divided into a severity score ranging from 0 (no symptoms or normal) to 3 (severe) as an ordinal, and the data were presented in form of distribution of the percentage of severity in the column chart.

In the comparison process, we used an unpaired T-test to analyze the differentiation of the severity of complications between two groups at different follow-up times, which were considered significant at *P* < 0.05 for a two-tailed test. Statistical analysis was performed using Microsoft Excel.

## Results

### Severity of skin color changes

**Figure 2:**
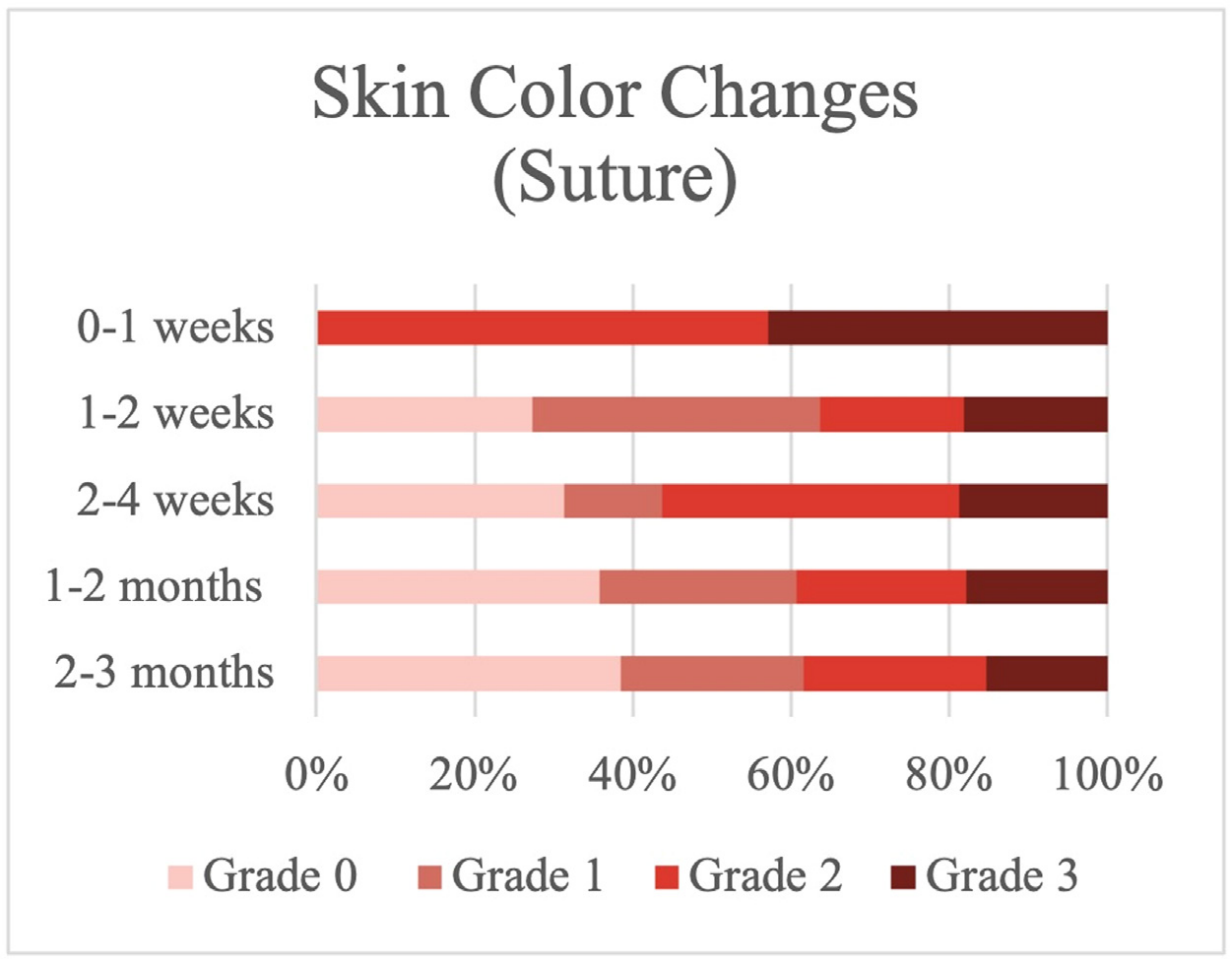
Skin color changes in the suture group.

**Figure 3:**
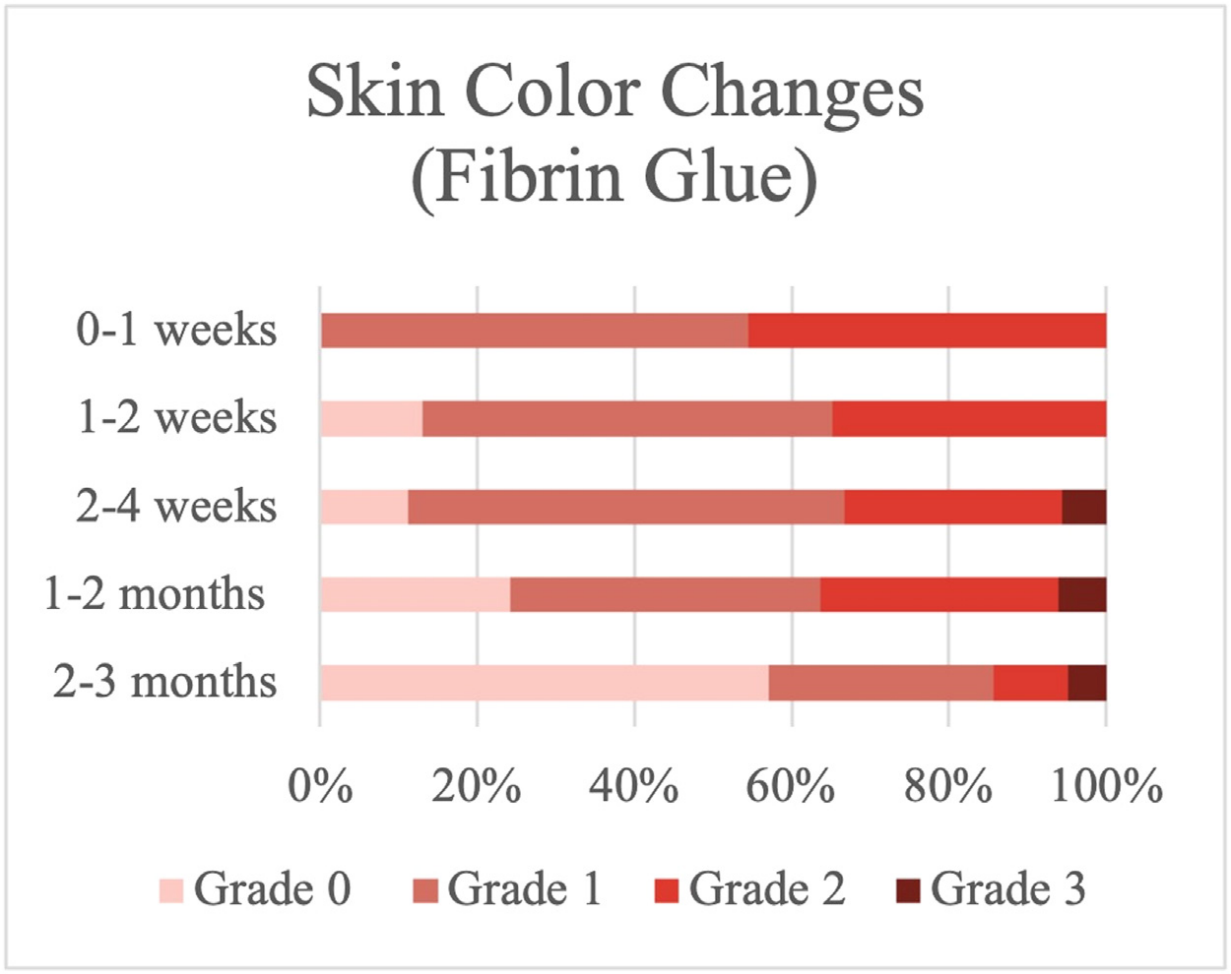
Skin color changes in the fibrin glue group.

Focusing on changes in skin color after surgery, we found that 57.14 % of the patients who underwent surgery via the suture procedure had purplish skin (Grade 2), and 42.85 % had red- or black-colored skin (Grade 3) in the first-week postoperation (Figure 2), whereas 54.54 % of the patients on whom fibrin glue was used had yellow skin (Grade1) and no one had Grade 3 skin changes in the first week (Figure 3). Using unpaired T-test analysis, the severity of skin color changes was significantly less with fibrin glue than with the suture technique (*P* value 0.001) (Table 2).

**Table 2:** Comparison of downtime scores for two techniques.

Complication outcomes, x̅ (N)	Surgical technique for anchoring transposition fat		
	Suture (*n* = 55)	Fibrin glue (*n* = 72)	*P* value
Changes in skin color			
0–1 week	2.43 (7)	1.45 (11)	0.001
1–2 wk	1.27 (11)	1.2 (15)	0.47
2–4 wk	1.44 (16)	1.27 (18)	0.38
1–2 months	1.21 (28)	1.18 (33)	0.87
2–3 months	1.15 (13)	0.62 (21)	0.1
Range of eyelid contusion			
0–1 week	2.57 (7)	1.82 (11)	0.1
1–2 wk	1.64 (11)	1.47 (15)	0.7
2–4 wk	1.63 (16)	1.83 (18)	0.6
1–2 months	1.32 (28)	1.39 (33)	0.81
2–3 months	1.15 (13)	0.62 (21)	0.15
Tension of eyelid swelling			
2 wk	0.5 (10)	0 (5)	0.05
4 wk	0.46 (49)	0.15 (69)	0.001

The *P*-value of 0.05 or less was judged to indicate a significant difference.

However, 1-week postoperation, there was no significant difference in the results of skin color change between the two groups (*P* value > 0.05).

### Severity of the range of skin contusion

**Figure 4:**
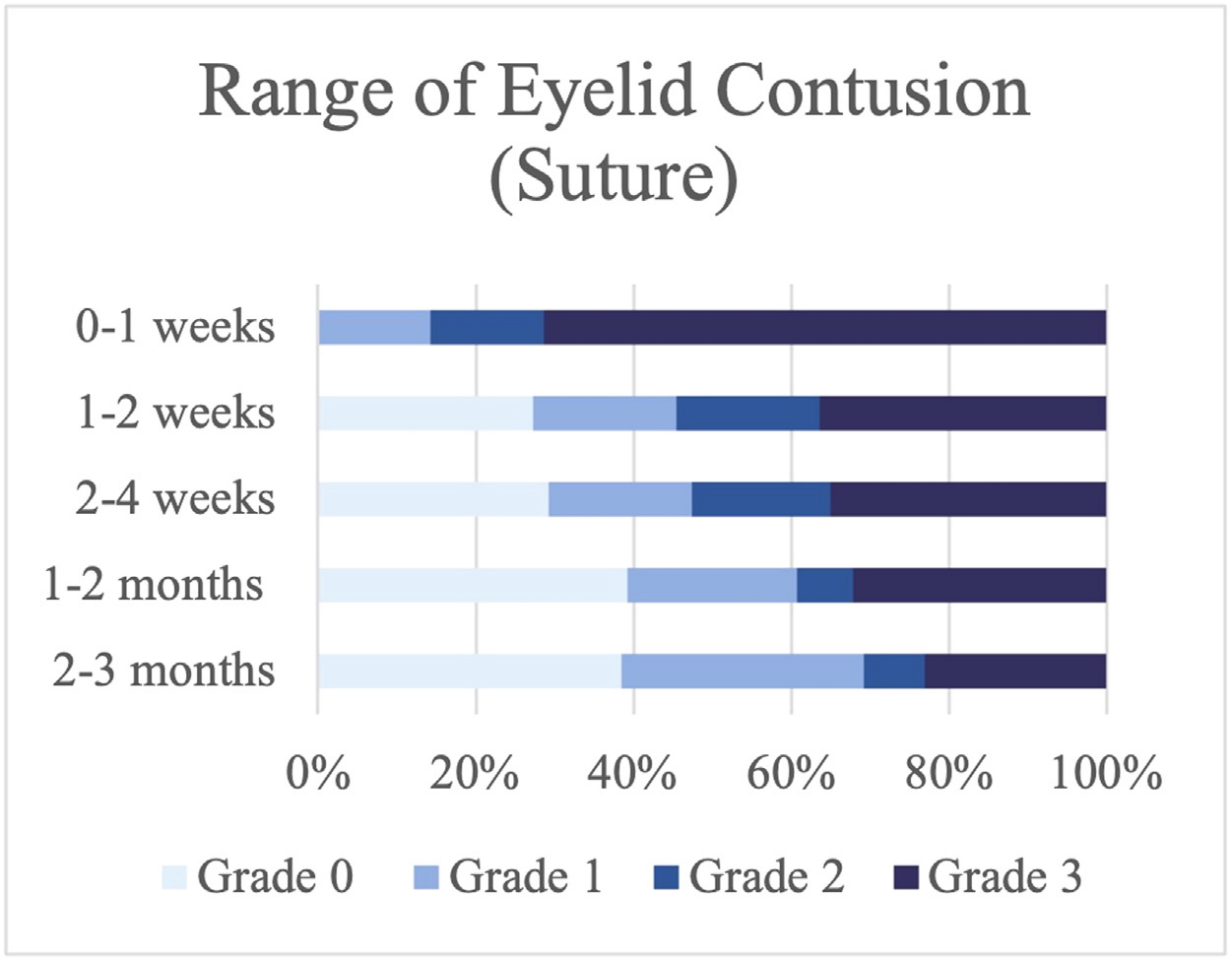
Range of skin contusion in the suture group.

**Figure 5:**
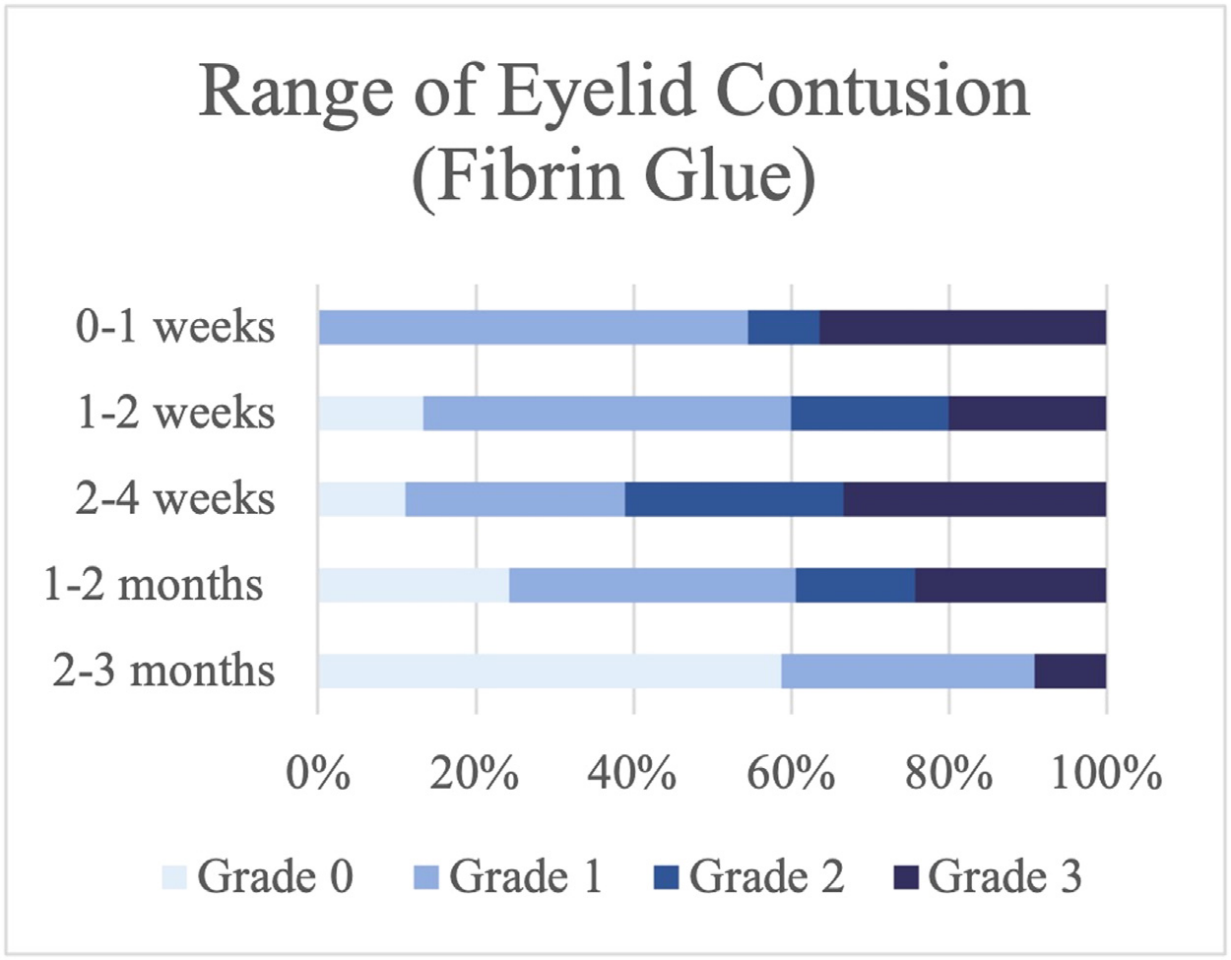
Range of skin contusion in the fibrin glue group.

Most patients who underwent the suture technique had Grade 3 eyelid contusion that extended to the upper eyelid in the first 2 wk, with 71.42 % experiencing this in the first week and 36.36 % in the second week (Figure 4). In contrast, only 36.36 % of the patients on whom fibrin glue was used had contusions that were as severe. Instead, most of the fibrin glue group had contusions that only involved the lower eyelid in the first week (54.54 %) and after 2 wk of surgery (46.67 %) (Figure 5).

However, the two techniques were not significantly different when analyzed at all follow-up times (*P* value > 0.05) (Table 2).

### Severity of tension in the skin

**Figure 6:**
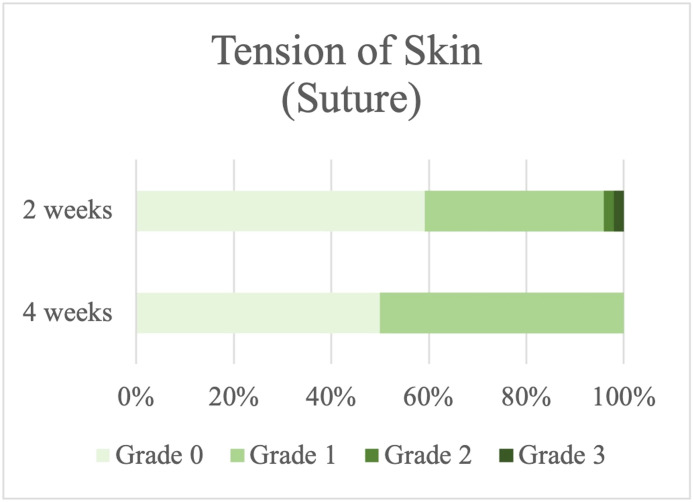
Skin tension in the suture group.

**Figure 7:**
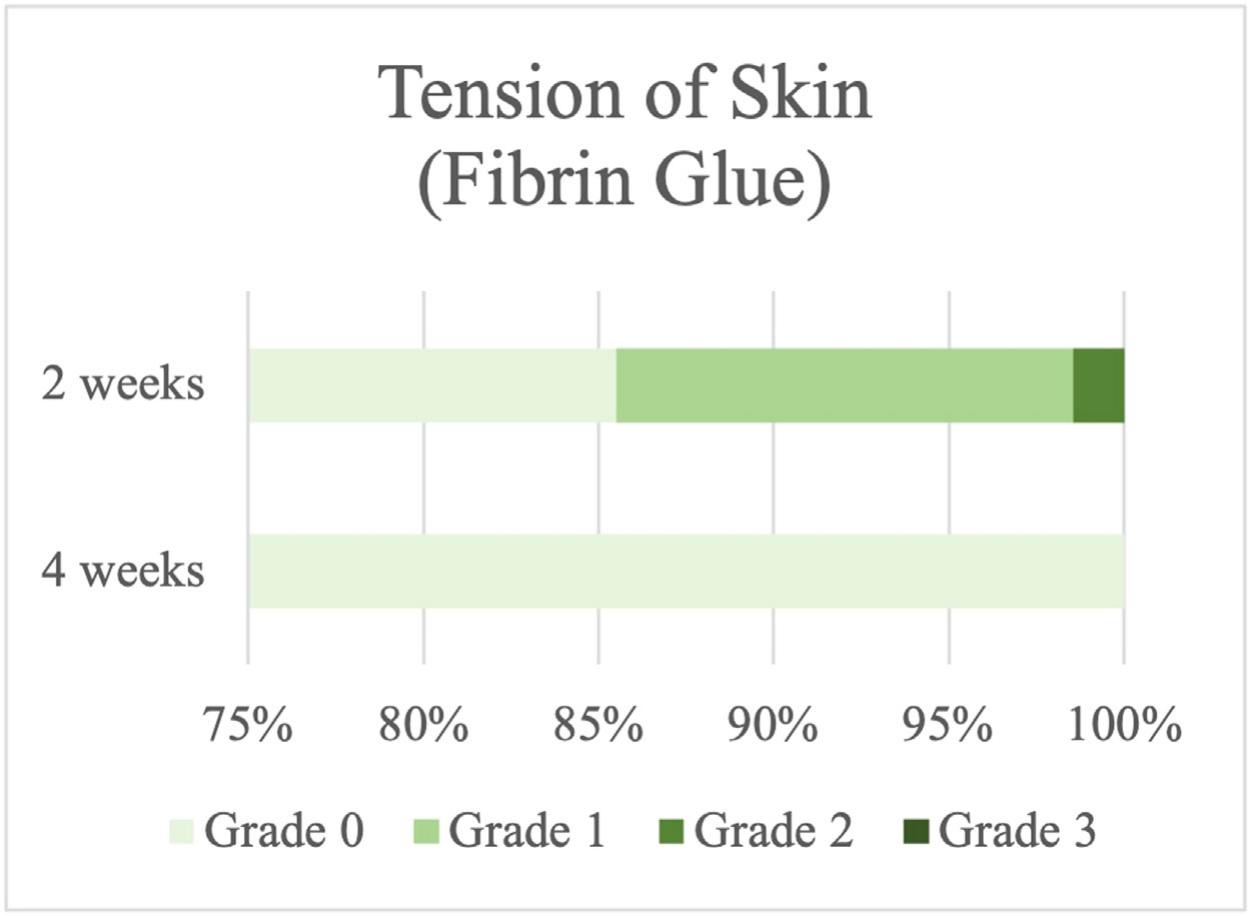
Skin tension in the fibrin glue group.

None of the patients who had fat anchored with fibrin glue experienced an MRD-2 change of >3 mm (Grade 3); in contrast, 2.04 % of the patients who were sutured had Grade 3 severity that was changed MRD-2 > 3 mm or suffered from difficulty in opening their eyelid (Figures 6, 7).

Comparing before and after the operation, patients who had fat anchored with fibrin glue had considerably less skin tension than those in the other group 2 wk after surgery, but their outcomes were also better at 1-month postoperation (*P* value 0.05, 0.001) (Table 2).

### Complications

In the suture group, there were two cases of ectropion, one case of conjunctival adhesion, one case of hematoma, and one case of infection out of the 55 cases. In the fibrin glue group, there were three cases of conjunctival adhesions, one case of ectropion, and one case of scar formation of the inferior oblique muscle out of the 72 cases.

## Discussion

In recent years, the use of fibrin glue has gradually acquired recognition in the field of ophthalmic surgery.^6^ For example, fibrin glue may cause less recurrence and require less time than sutures for fixing the conjunctival graft in place during pterygium surgery.^7^ Moreover, fibrin glue is used for the closure of blepharoplasty incisions.^8^ When human tissue is injured, bleeding ensues and then ceases due to blood clot formation. This is the initial mechanism of natural wound closure. A clot is a product of the final common pathway of blood coagulation. Fibrin glue mimics this coagulation cascade, resulting in its adhesive capability.^9^

In this retrospective study, we found that the use of fibrin glue resulted in less postoperative complication than the suture technique, particularly during the first month after surgery. This result in minimal changes to skin color after the operation, which may be seen as a lighter shade of skin bruising or yellow due to the hemostatic effect of fibrin glue that reduces bleeding.

Periosteal fixation, although commonly used in lower eyelid blepharoplasty to anchor fat pedicles, has several limitations, including technical difficulty and prolonged operative time. Periosteal fixation requires precise dissection and suturing to the periosteum, which increases the complexity of the procedure and may lead to longer recovery times. In contrast, fibrin glue offers a simplified approach without the need for extensive manipulation, reducing surgical duration and patient recovery time.

Moreover, the use of fibrin glue prevents increasing tension in the surgical wound, which was common when sutures are used, so that it may reduce swelling and skin tension after the operation. Consequently, there was a significant decrease in the change of MRD-2 pre- and postoperatively compared to the suture technique. We believe that the postoperative MRD-2 changes, in this case, were mainly due to swelling, but a previous report showed that MRD-2 decreased after lower eyelid transconjunctival blepharoplast,^10^ and we cannot exclude this effect. However, the fact that there was a significant difference between the two groups without changing the method of approach suggests that fibrin glue promotes hemostasis and reduces postoperative swelling.

However, the range of skin contusion was not differentiated between the two groups because this condition resulted from trauma during the surgical process, including the incision process and fat repositioning, which was performed on both groups.

The absence of external sutures with fibrin glue fixation significantly contributes to reduced social downtime, enabling patients to return to daily and social activities more comfortably. This benefit has notable implications for the patient's quality of life, as it reduces the visible signs of surgery and enhances postoperative recovery satisfaction. The primary endpoint, time to social downtime recovery, highlighted the advantages of fibrin glue in minimizing the social and psychological burden of visible sutures on patients, facilitating a faster return to normal social interactions without the concern of visible sutures impacting the appearance.

Although most patients who underwent suture techniques had more severe downtime than the other group, there was no significant difference in the results 1-month postoperation.

The frequency of complications and recurrence did not differ significantly between the two groups.

## Conclusion

Among the patients who underwent transconjunctival lower eyelid blepharoplasty with fat transposition, the use of fibrin glue produced a substantially lower severity of skin coloring changes and tension of hematoma in the first few weeks of postoperation compared to the sutures. However, there was no significant difference in the outcomes after 1 month of surgery. There was no significant difference in the frequency of complications between the two groups.

## Declaration of competing interest

The authors declare that they have no competing interests or financial disclosures to report.
